# Mechanisms and recent advances in the diagnosis and treatment of nitrous oxide-induced peripheral neuropathy: a narrative review

**DOI:** 10.3389/fneur.2024.1381938

**Published:** 2024-05-23

**Authors:** Xiaodi Zou, Fangyu Yi, Weijie Zhou, Yanzhao Dong, Ahmad Alhaskawi, Haiying Zhou, Sohaib Hasan Abdullah Ezzi, Vishnu Goutham Kota, Mohamed Hasan Abdulla Hasan Abdulla, Olga Alenikova, Sahar Ahmed Abdalbary, Hui Lu, Changxin Wang

**Affiliations:** ^1^Department of Orthopedics, The Second Affiliated Hospital of Zhejiang Chinese Medical University, Hangzhou, Zhejiang, China; ^2^The First School of Clinical of Zhejiang Chinese Medical University, Hangzhou, Zhejiang, China; ^3^Department of Orthopedics, No. 903 Hospital of PLA Joint Logistic Support Force, Hangzhou, China; ^4^Department of Orthopedics, The First Affiliated Hospital, Zhejiang University, Hangzhou, Zhejiang, China; ^5^School of Biomedical Sciences, Faculty of Medicine, The Chinese University of Hong Kong, Shatin, Hong Kong SAR, China; ^6^Department of Orthopedics, Third Xiangya Hospital, Central South University, Changsha, Hunan, China; ^7^Zhejiang University School of Medicine, Hangzhou, Zhejiang, China; ^8^Department of Neurology, Republican Research and Clinical Center of Neurology and Neurosurgery, Minsk, Belarus; ^9^Department of Orthopedic Physical Therapy, Faculty of Physical Therapy, Nahda University in Beni Suef, Beni Suef, Egypt

**Keywords:** nitrous oxide – N_2_O, peripheral neuropathy, abusive inhalation, neurological disorders, clinical characteristics

## Abstract

Under standard conditions, nitrous oxide (N_2_O) manifests as a colorless, odorless gas with a mildly sweet taste. The compound finds applications in various fields, including its use as an aerosol propellants, an accelerant in motor racing, and an anesthetic in surgical procedures and dentistry. Unfortunately, the recreational misuse of N_2_O has become prevalent among young individuals due to its euphoric and hallucinogenic effects. Compounding this issue is the fact that nitrous oxide can be easily obtained from over-the-counter household items, facilitating its non-medical use. The global community has witnessed a surge in the recreational utilization of nitrous oxide gas in recent years. Despite the widespread non-medical abuse of N_2_O, there remains inadequate understanding of the potential adverse effects resulting from exposure to it. This paper provides an overview of management findings, laboratory and electrodiagnostic characteristics, as well as clinical presentations associated with neurological disorders induced by nitrous oxide usage.

## Introduction

N_2_O is primarily utilized as an anesthetic agent. Distinguished from other inhalants, the inhalation of nitrous oxide induces a profound, transient, and pleasurable euphoria that is often described as mildly psychedelic and agreeable. It also subtly alters body image perception and can result in sensations of dissociation (1) along with its evanescent effects and rapid restoration of normal faculties, recreational users highly desire N_2_O for brief intoxication purposes, typically experiencing its effects within minutes. In recent years, the euphoric properties of N_2_O have led to widespread recreational use in the Western world (2). For instance, data collected from drug users across more than 30 countries through the 2019 Global Drug Survey (GDS) revealed that at least once in their lifetime, 90% of respondents had used N_2_O, positioning it as the tenth most popular substance in Western society after alcohol and tobacco.

The utilization patterns of N_2_O are similar across these nations with a particular prevalence of ‘whippets’—small canisters containing the gas. However, it is crucial to note that chronic exposure to elevated doses of N_2_O can result in significant neurological damage including cobalamin (vitamin B_12_) deficiency-induced neuropathy and even paralysis.

Therefore, it is imperative to understand and address the potential risks associated with prolonged inhalation of N₂O (3, 4). Additionally, the increasing incidence of individuals presenting at emergency departments with neurological impairments due to N₂O exposure highlights the concerning and serious nature of this trend (5).

Many of case reports have firmly established a clear correlation between the misuse of N₂O and a range of neurological and psychiatric disorders, including conditions such as subacute combined degeneration of the spinal cord, myelopathy, demyelinating polyneuropathy, peripheral neuropathy, and various mood and affective disturbances (6–8). Furthermore, fatalities resulting from N₂O inhalation have been documented (9). Currently, only a limited number of studies have focused on the peripheral neuropathy caused by abusive inhalation of nitrous oxide (10–12), highlighting a lack of awareness regarding the toxicity associated with N₂O abuse. Therefore, this study aims to comprehensively outline the clinical characteristics, mechanisms, and management strategies for N₂O-associated peripheral neuropathy.

## The mechanisms of N_2_O neurotoxicity

The manifestation of peripheral neuropathy can occur through various mechanisms, including distal axonopathy, myelinopathy, and neuronopathy. Each mechanism involves distinct pathological processes that result in the degeneration or dysfunction of nerve fibers, thereby impairing their ability to effectively transmit signals (13–15).

To date, Vitamin B_12_, as called cobalamin, insufficiency has been extensively researched, while the exact mechanism of N_2_O remains unclear. Although it has been observed that individuals who persistently use N_2_O and experience neurological damage tend to have lower cobalamin levels, it is doubtful that a vitamin B_12_ shortage is the sole cause of such damage (11). Elevated serum levels of methylmalonate and homocysteine have proven to be more reliable biomarkers for brain injury following prolonged exposure to N_2_O (16), promoting further investigation into the specific metabolic pathways underlying the toxicity associated with N_2_O exposure.

Cobalamin, also known as Vitamin B_12_, contains a cobalt ion at its core. Within the human body, it exists in two biologically active forms: methylcobalamin and adenosylcobalamin. Under specific conditions, N_2_O can induce neurotoxic effects primarily through various biochemical mechanisms. One significant pathway of neurotoxicity involves interference with Vitamin B_12_ metabolism. N_2_O oxidizes the cobalt ion from its functional +1 oxidation state to a non-functional +3 state (11). This oxidation renders cobalamin ineffective as a coenzyme for methionine synthase and methylmalonyl-CoA mutase (MMCoAM), thereby disrupting critical cellular processes such as DNA synthesis and energy production. The clinical implications of this disruption may include neurological dysfunction and hematological disorders due to impaired methionine synthesis and accumulation of homocysteine and methylmalonic acid (11). Furthermore, deficiency in MMCoAM enzymatic activity during lipid and carbohydrate biosynthesis leads to intracellular accumulation of methylmalonate acid (17, 18).

Another pathway involves methionine methyltransferase (MTR), a pivotal enzyme responsible for catalyzing the conversion of homocysteine and 5-methyltetrahydrofolate into tetrahydrofolate and methionine. Consequently, insufficient MTR enzymatic activity may result in an accumulation of homocysteine and 5-methyltetrahydrofolate, accompanied by reduced levels of methionine, tetrahydrofolate, and *S*-adenosylmethionine (19). Impairment in methionine and *S*-adenosylmethionine synthesis can disrupt the methylation process of myelin phospholipids, leading to various neurological consequences such as demyelination in the brain, spinal cord, and peripheral nervous system. Clinically, this disruption may manifest as megaloblastic anemia with potential progression to optic nerve atrophy (20, 21). Meanwhile, elevated levels of homocysteine can exert detrimental effects on physiological systems through distinct pathways: induction of oxidative stress resulting in reactive oxygen species (ROS) generation that triggers apoptotic cell death; activation of NMDA receptors (22). The activation of NMDA receptors has the potential to increase extracellular Ca^2+^ influx, cause mitochondrial Ca^2+^ overload and dysfunction along with ROS formation, potentially serving as the primary mechanism underlying homocysteine-mediated neurotoxicity (22).

Vitamin B_12_ depletion is not the only factor contributing to the neurotoxic effects observed after exposure to nitrous oxide (N_2_O); other substantial mechanisms are also involved. Neonatal cerebral structures are especially vulnerable to N_2_O-induced neurotoxicity, which occurs through antagonism of *N*-methyl-d-aspartate (NMDA) receptors (22). The activation dynamics of NMDA antagonists are widely recognized to produce divergent effects, ranging from neuroprotection to neurotoxicity (22). Short-term exposure to N_2_O can cause reversible vacuolization in neuronal cells, while prolonged exposure is associated with neuronal apoptosis. Importantly, vacuolization involves significant swelling of mitochondrial structures (23).

**Figure 1:**
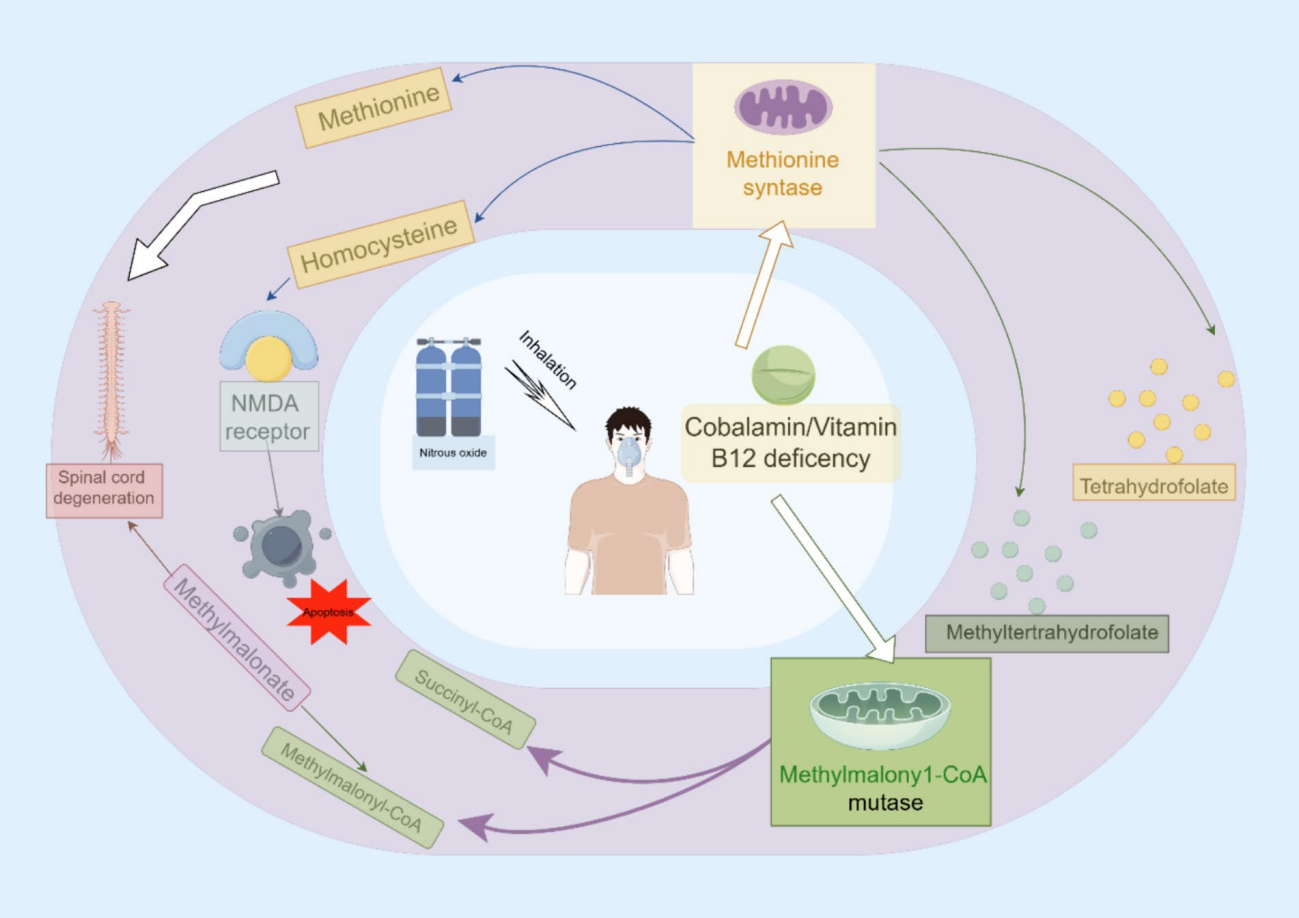
The main mechanisms involved in N_2_O-induced neurotoxicity.

It has been suggested that a change in cerebral blood flow is one of the underlying mechanisms responsible for the neurotoxic ramifications of N_2_O, particularly in terms of cerebral damage (22). Furthermore, N_2_O alone can inhibit the biosynthesis of xanthine and various monoamines, such as norepinephrine, dopamine, and serotonin. This inhibition may lead to neurotoxic outcomes, subsequently triggering a cascade of events including cytokine disequilibrium, cerebral hypoxia, and acidosis (Figure 1) (24).

## Clinical features

The symptoms of peripheral neuropathy vary depending on the location and type of nerve damage. Common manifestations include paresthesia in the hands and feet, muscle weakness or paralysis, impaired balance or coordination, as well as pain (25).

Abuse of N_2_O can lead to peripheral neuropathy, a condition marked by symptoms such as weakness, numbness, and unsteady gait (26). Notably, the weakness is more pronounced in the lower limbs compared to the upper limbs. Studies of nerve function have consistently demonstrated that this nerve damage involves both motor and sensory fibers, with a considerable loss of motor nerve axons in the lower extremities (26). Further investigations, including sural nerve biopsies, have confirmed that ongoing axonal degeneration is the primary pathological change in nerves affected by this condition (26, 27).

A retrospective study (28) spanning was conducted between 2018 and 2020, involving 76 patients diagnosed with neuropathy attributable to N_2_O misuse. The analysis of the collected data indicated that 36% of these patients exhibited an absence of response in nerve conduction assessments. Notably, the majority presented with reduced sensory and motor nerve conduction velocities, affecting 75 and 76% of the cohort, respectively. Additionally, diminished amplitudes in sensory nerve action potentials and compound muscle action potentials were observed in 57 and 59% of cases, respectively, along with prolonged distal motor latencies. The electrophysiological data (28) revealed diverse neuropathic presentations, with axonal neuropathy identified in 37 patients (49%), demyelinating peripheral neuropathy in 4 patients (5%), and mixed neuropathy in 12 patients (16%). The primary pathological features included predominant motor axonal damage in 67% of the upper and lower limb impairments, and sensory nerve demyelination accounting for 35% of the deficits. Furthermore, a subgroup analysis suggested a correlation between prolonged N_2_O exposure, extended illness duration, and the severity of motor axonal damage in the lower extremities.

In our case series, the nerve conduction studies of a typical patient with peripheral neuropathy induced by N_2_O revealed complaints of fatigue and numbness in the bilateral lower limbs (Tables 1, 2) (1, 2).

**Table 1:** Motor nerves conduction.

Nerve and site	Latency	Amplitude	Velocity
Peroneal. R			
Ankle	4.9 ms	0.8 mV	N/A
Fibula (head)	12.2 ms	0.1 mV	12.3 m/s
Tibial. L			
Fibula (head)	5.0 ms	2.5 mV	N/A
Popliteal fossa	14.3 ms	1.1 mV	41.3 m/s
Peroneal. L			
Ankle	6.4 ms	0.4 mV	N/A
Fibula (head)	14.3 ms	0.2 mV	40.5 m/s
Popliteal fossa	16.0 ms	0.1 mV	50.5 m/s
Tibial. R			
Fibula (head)	5.0 ms	0.7 mV	N/A
Popliteal fossa	14.7 ms	0.1 mV	40.2 m/s
Median. R			
Wrist	4.3 ms	3.6 mV	N/A
Below elbow	9.0 ms	2.7 mV	51.4 m/s
Ulnar. R			
Wrist	3.0 ms	7.6 mV	N/A
Below elbow	7.9 ms	5.3 mV	40.8 m/s
Above elbow	10.0 ms	7.2 mV	54.7 m/s
Axilla	11.2 ms	7.0 mV	60.0 m/s
Median. L			
Wrist	4.4 ms	4.0 mV	N/A
Below elbow	8.6 ms	2.6 mV	53.5 m/s
Ulnar. L			
Wrist	2.9 ms	10.7 mV	N/A
Below elbow	6.8 ms	10.7 mV	53.8 m/s
Above elbow	9.0 ms	10.7 mV	50.0 m/s
Axilla	10.3 ms	10.7 mV	53.8 m/s

^*^R means right side limb. L means left side limb.

**Table 2:** Sensory nerves conduction.

Nerve and site	Latency	Amplitude	Velocity
Sural. R			
Fibula (head)	2.3 ms	2.9 mV	41.3 m/s
Superficial peroneal. R			
Fibula (head)	2.2 ms	8.5 mV	43.1 m/s
Sural. L			
Fibula (head)	2.3 ms	4.6 mV	43.1 m/s
Superficial peroneal. L			
Fibula (head)	2.4 ms	3.4 mV	43.7 m/s
Superficial of ulnar. R			
Digit V	2.5 ms	8.1 μV	46.0 m/s
Superficial of ulnar. L			
Digit V	2.4 ms	9.4 μV	50.0 m/s

^*^R means right side limb. L means left side limb.

## Diagnosis

In clinical practice, several diagnostic tests are available to identify the underlying etiology and assess the extent of peripheral nerve damage. Commonly employed techniques include nerve conduction studies (NCS), electromyography (EMG), imaging modalities, and nerve biopsy (29). When assessing patients, especially younger individuals, who exhibit symptoms indicative of peripheral neuropathy or myelopathy, clinicians should contemplate the potential for N_2_O neurotoxicity. A detailed history of specific and prolonged N_2_O use and exposure is essential for diagnostic confirmation. However, it is important to note that some patients may not disclose their N_2_O usage during initial consultations, which can complicate the process of establishing a preliminary diagnosis. Moreover, Guillain-Barré syndrome (GBS) and N_2_O-related peripheral neuropathy share several similarities (11), necessitating additional biochemical testing and nerve conduction testing to be conducted (30).

Biochemical testing for functional vitamin B_12_ insufficiency, such as accessing homocysteine and methylmalonic acid levels, can be used to confirm the diagnosis in cases where there are consistent clinical symptoms and a history of significant N_2_O exposure (Table 1) (31). Furthermore, it is recommended to conduct nerve conduction studies in order to further characterize the involvement of the peripheral nervous system (Table 3) (32, 33).

**Table 3:** Diagnostic examinations for individuals with suspected nitrous oxide poisoning (31).

Investigation type	Finding
Vitamin B_12_	Patients with neurologic symptoms often have either low (50–75%) or normal (25–50%) levels.
Homocysteine	Increased
Methylmalonic acid	Increased
Nerve conduction studies	The majority of patients with symptoms exhibit abnormality. Axonal degeneration, with or without demyelination, is a common occurrence. Isolated demyelination without axonal degeneration is a rare phenomenon.

A low concentration of vitamin B_12_ is observed in 54–72% of patients experiencing neurological issues due to N_2_O exposure (34, 35). This occurrence is more likely in individuals exhibiting symptoms after shorter doses, indicating increased susceptibility (35). Low concentrations of vitamin B_12_ in long-term users may be indicative of accelerated clearance (36, 37). This reduced enzymatic activity results in the accumulation of homocysteine and methylmalonic acid, with at least one of these being elevated in over 90% of patients (38). Consequently, these biomarkers are more sensitive indicators compared to vitamin B_12_ concentrations, as the latter can remain within the normal range in a significant proportion of users despite neurotoxicity. In an attempt to mitigate neurotoxicity and maintain normal levels of these biomarkers, some users supplement with additional vitamin B_12_. However, although this practice may potentially mislead clinicians, it does not offer complete protection against the neurological side effects associated with nitrous oxide usage (39, 40).

The majority of patients exhibiting symptoms demonstrate atypical results in nerve conduction studies (33–35). While a minority of these individuals exhibit signs of isolated demyelination, the predominant irregularity observed is axonal degeneration, which may occur with or without accompanying demyelination. It is noteworthy that individuals who regularly use N_2_O tend to experience more pronounced motor impairments compared to those with a deficiency of vitamin B_12_ not associated with nitrous oxide exposure (41).

**Figure 2:**
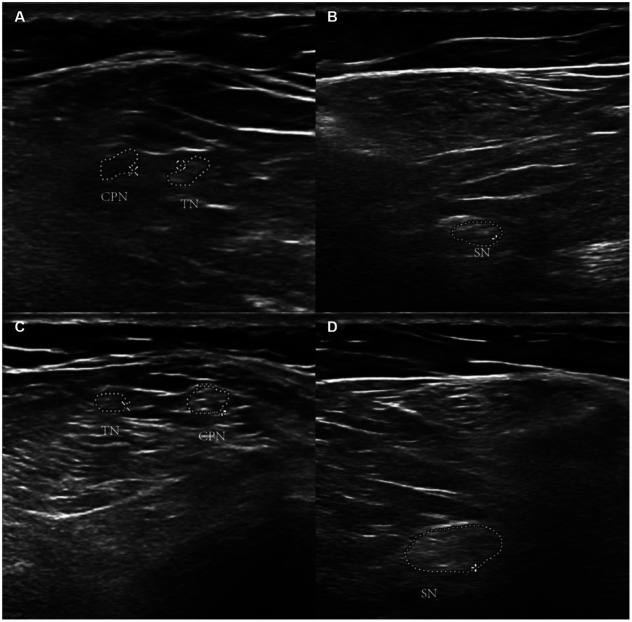
Ultrasonographic image of nitrous oxide-Induced peripheral neuropathy. Panels **(A,B)** show the echo enhancement around peripheral nerves in the left lower limb. Panels **(C,D)** are the right lower limb. ^*^TN refers to the tibial nerve. CPN refers to common the peroneal nerve. SN refers to the sciatic nerve.

In previous studies, MRI was used to diagnose lesions on the spinal cord and cerebral cortex (27). Based on our expertise, we recommend using ultrasonography to identify peripheral nerve impairments (42, 43). Echo enhancement around peripheral nerves can be observed with ultrasound (Figure 2).

In conclusion, when patients with a history of N_2_O use present symptoms of peripheral neuropathy, physicians should consider the possibility of N_2_O-induced peripheral neuropathy. Further nerve conduction studies (NCS) can confirm the presence of peripheral nerve damage. Additionally, a concurrent decrease in Vitamin B_12_ levels can aid in diagnosing N_2_O-related peripheral neuropathy.

## Treatment

Prior research has indicated that prolonged usage of N_2_O heightens the likelihood of neurological impairments, and discontinuing exposure to the toxin is the foremost crucial initial measure for treatment (11, 12, 30). Supplementation with vitamin B_12_ is advised, and in some cases, it may be combined with methionine, despite the limited evidence underpinning its efficacy (31). We propose an administration of 1,000 μg of vitamin B_12_, either subcutaneously or intramuscularly, on a daily basis for 1–2 weeks. Subsequently, either weekly injections administered by caregivers or daily oral doses of 2,000 μg should continue until symptomatic relief is achieved. This recommendation is based on the favorable safety profile of vitamin B_12_ (44, 45). Furthermore, we propose a secure and efficacious regimen of methionine supplementation, with an oral dosage of 1 g administered three times daily for a minimum of 4–6 weeks, or until significant symptomatic improvement is observed (46). Due to the potential for exacerbation of symptoms and prolonged recovery, initiating folate supplementation before the restoration of vitamin B_12_ levels is not recommended, as it is unlikely to benefit the patient (47, 48). In certain cases, integrating physical rehabilitation along with social and psychological support measures may be essential.

The beneficial effects of hyperbaric oxygen therapy (HBOT) in repairing peripheral nerve injuries have been well-documented in previous literature (49). A recent prospective study (50) assessed the efficacy of HBOT following primary nerve repair in patients with upper extremity nerve injuries. The study results shown that compared to the control group, the group treated with hyperbaric oxygen achieved a higher power score, exhibited a faster recovery rate, and demonstrated quicker impulse transmission. However, there is limited documentation regarding the use of HBOT for treating peripheral nerve injuries caused by N_2_O. In our experience, we are investigating the potential use of HBOT as an adjunctive treatment for patients with peripheral neuropathy induced by the abusive inhalation of N_2_O.

**Figure 3:**
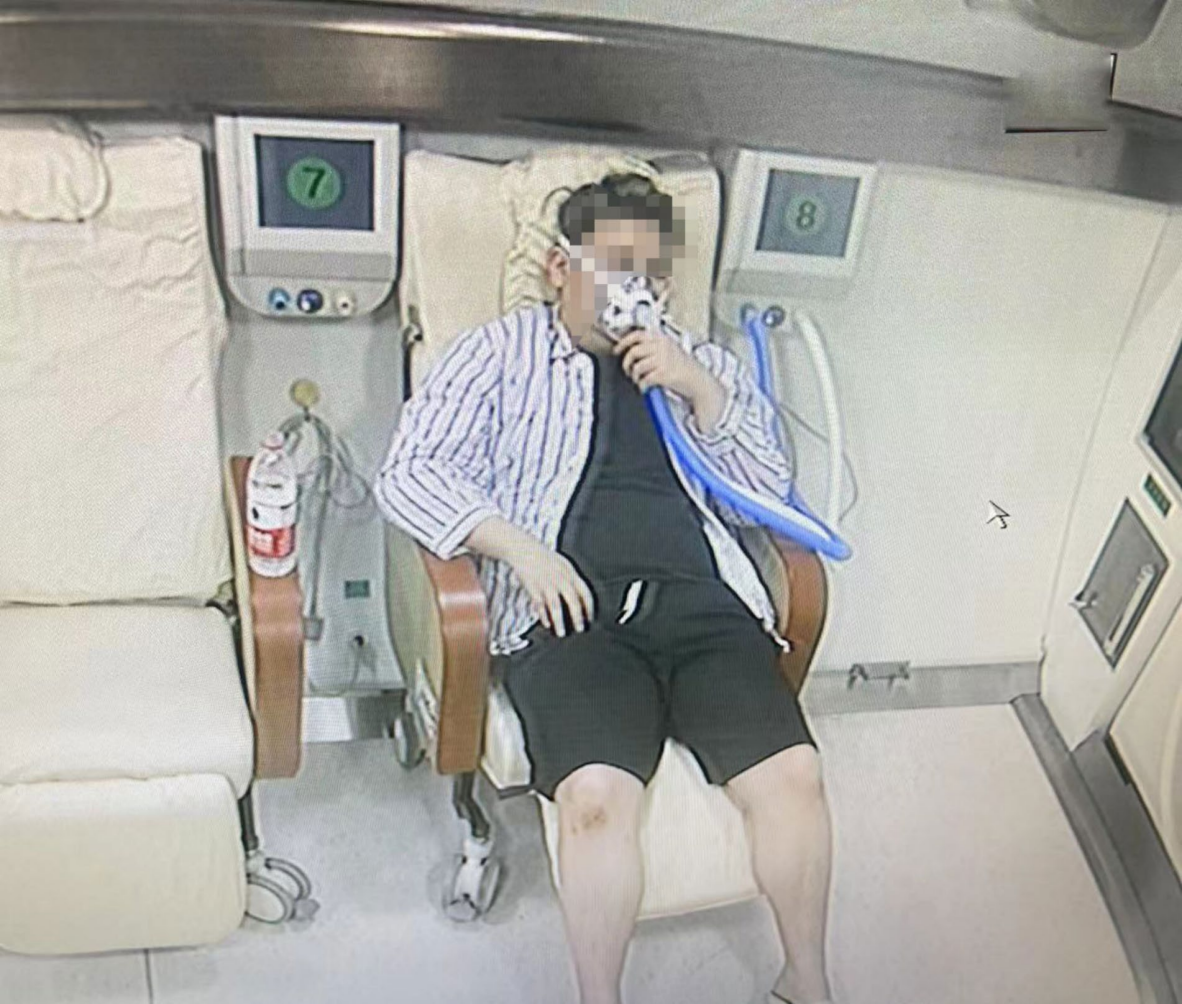
A patient with peripheral neuropathy caused by excessive inhalation of nitrous oxide is undergoing treatment with hyperbaric oxygen therapy.

The prognosis for recovery varies among patients; however, the vast majority of patients (95–97%) exhibit some degree of improvement. It is important to note that despite months of therapeutic intervention, over one-third of hospitalized patients continue to manifest neurological symptoms (Figure 3 and Table 4) (34, 35).

**Table 4:** Treatment for peripheral neuropathy caused by N_2_O.

Methods	Description
Cessation of exposure	This represents the initial stage. Consideration should be given to specialized knowledge in addiction medicine, along with the provision of psychiatric, psychological, and social support.
B_12_ (cobalamin)	Administer 1,000 micrograms intramuscularly on a daily basis for a period of 1–2 weeks, then switch to a weekly dosage of 1,000 micrograms or a daily oral dosage of 2,000 micrograms until symptoms are resolved.
Methionine	1 g by mouth 3 times a day for at least 4–6 weeks, or until symptoms get a lot better.
Other	1.Rehabilitation. 2.Do not give folate before giving B_12_ supplements. 3.Hyperbaric oxygen therapy.

## Conclusion

Nitrous oxide, is known for its cost-effectiveness and ease of procurement. It is widely utilized as a recreational substance, particularly among the adolescent population. Its consumption is a frequently overlooked as a potential cause of neurological disorders, primarily myelopathy, peripheral neuropathy, and encephalopathy, which may be accompanied by hematological abnormalities. Furthermore, it has the potential to induce functional vitamin B_12_ deficiency. Therefore, healthcare professionals are encouraged to consider and inquire about nitrous oxide use in patients presenting with unexplained clinical manifestations suggestive of vitamin B_12_ deficiency or other neurologic symptoms consistent with its usage.

In conclusion, a comprehensive understanding and recognition of the neurological implications associated with the utilization of N_2_O is imperative for healthcare professionals. By considering the potential involvement of N_2_O in patients presenting with inexplicable neurological symptoms or exhibiting signs of vitamin B_12_ deficiency, healthcare professionals can assume a pivotal role in early detection, diagnosis, and management of related conditions, thereby enhancing patient care and optimizing outcomes.

## Author contributions

XZ: Writing – original draft, Writing - review & editing. FY: Writing – original draft, Writing – review & editing. WZ: Visualization, Writing – review & editing. YD: Investigation, Writing – review & editing. AA: Funding acquisition, Writing – review & editing. HZ: Methodology, Writing – review & editing. SE: Software, Writing – review & editing. VK: Supervision, Writing – review & editing. MA: Validation, Writing – review & editing. OA: Data curation, Formal analysis, Writing – review & editing. SA: Software, Writing – review & editing. HL: Conceptualization, Writing – review & editing. CW: Conceptualization, Writing – review & editing.
